# Partial replacement of soybean meal with full-fat black soldier fly larvae meal in plant-based nursery diets did not influence fecal *Escherichia coli* colony forming units or improve fecal consistency when pigs were weaned into non-disinfected pens

**DOI:** 10.1093/tas/txad121

**Published:** 2023-10-20

**Authors:** B Christensen, C Zhu, M Niazy, T McCullough, N Ricker, L Huber

**Affiliations:** Department of Animal Biosciences, University of Guelph, Guelph, ON N1G 2W1, Canada; Department of Animal Biosciences, University of Guelph, Guelph, ON N1G 2W1, Canada; Department of Pathobiology, University of Guelph, Guelph, ON N1G 2W1, Canada; Department of Pathobiology, University of Guelph, Guelph, ON N1G 2W1, Canada; Department of Pathobiology, University of Guelph, Guelph, ON N1G 2W1, Canada; Department of Animal Biosciences, University of Guelph, Guelph, ON N1G 2W1, Canada

**Keywords:** black solider fly larvae meal, cecal mucosal microbial profile, fecal score, growth performance, nursery pig

## Abstract

At weaning, one hundred pigs (21 d of age; 6.96 ± 0.23 kg BW) were used to determine the effect of partially replacing soybean meal (SBM) in corn- and SBM-based nursery diets on growth performance, fecal scores, Escherichia coli (*E. coli)* colony forming units (CFU), and cecal mucosal microbial profile when weaned into non-disinfected nursery pens. Pens were randomly assigned to one of four dietary treatments (*n* = 5): high-complexity (contained highly digestible animal proteins and 10.8% SBM) with and without 3,000 ppm ZnO (HC **+** and HC−, respectively; representative of commercial diets), low-complexity (corn- and SBM-based; 31.8% SBM; LC), or LC with 30% inclusion of full-fat black soldier fly larvae meal (BSFLM) to partially replace SBM (LCFL; 8.0% SBM). Diets were fed for 14 d (phase I), followed by 4 wk of a common corn-SBM diet (phase II). Fecal *E. coli* CFU and cecal mucosal microbial 16s rRNA community profiles were assessed 7 d after weaning. During phase I, pigs fed LC and LCFL had lower average daily gains (*P* < 0.05) than pigs fed HC + and HC−, which were not different. Average daily feed intake was not different for pigs fed LC and LCFL, but lower than for pigs fed HC− (*P* < 0.001); pigs fed HC + had greater feed intake in phase I vs. all other treatment groups (*P* < 0.001). Upon nursery exit, only pigs fed LCFL had lower BW than pigs fed HC− (*P* < 0.05), with intermediate values observed for HC + and LC. Day 3 fecal scores were greater for pigs fed LCFL vs. HC + (*P* < 0.05) and day 7 *E. coli* CFU were greater for all treatment groups vs. HC + (*P* < 0.001). Pigs fed HC− (*P* < 0.01), LC (*P* < 0.05), and LCFL (*P* < 0.05) had lower alpha diversity for cecal mucosal microbiota compared to HC+. At the genus level, pigs fed LC had lower *Lactobacillus* relative abundance vs. pigs fed HC + (*P* < 0.01). Therefore, BSFLM can partially replace SBM without sacrificing growth performance vs. nursery pigs fed corn- and SBM-based diets, but both groups had reduced phase I growth performance vs. pigs fed highly digestible diets containing animal proteins when weaned into non-disinfected pens. The BSFLM did not influence fecal *E. coli* CFU or improve fecal consistency after weaning and therefore, is less effective at minimizing digestive upsets vs. HC + diets.

## Introduction

Weaning results in abrupt changes in environment, diet, and pathogen exposure that contribute to reductions in feed intake and growth for pigs (Pluske, 2016). Low feed intake for pigs directly following weaning also increases susceptibility to enteric disease, typically caused by the binding and proliferation of opportunistic bacteria such as Escherichia coli (*E. coli;*Heo et al., 2013). Historically, in-feed antibiotics or pharmacological levels of ZnO have been included in nursery diets to alleviate reduced growth performance associated with the post-weaning period (Thacker, 1999). However, the use of in-feed antibiotics as growth promoters has been banned in many countries due to concerns regarding antibiotic resistance, and the use of pharmacological levels of ZnO (>150 ppm) was recently banned in the European Union (in EMA, 2017; June 2022).

Typically, nursery diets include a variety of expensive, animal-based protein sources that a young pig can readily digest (e.g., whey, blood plasma meal, fish meal; Koo et al., 2020), while high inclusion of less expensive plant-based protein sources (e.g., soybean meal [SBM]) is generally precluded due to anti-nutritional factors (e.g., antigenic compounds, trypsin inhibitors, and fiber contents) that contribute to additional gastrointestinal dysfunction after weaning (Li et al., 1991; Chen et al., 2011). Conversely, full-fat black soldier fly larvae meal (*Hermetica illucens*; BSFLM) is a highly digestible protein and energy source for pigs (Crosbie et al., 2020) with a lower environmental footprint than SBM (Wang and Shelomi, 2017; Kim et al., 2019). Moreover, BSFLM contains medium-chain fatty acids (MCFA; specifically, lauric acid), antimicrobial peptides, and chitin, which may impart functional benefits to replace in-feed antibiotics and pharmacological levels of ZnO. Specifically, lauric acid provides antimicrobial and anti-inflammatory effects in the small intestine in part through bacteriostatic effects on both Gram-negative and Gram-positive bacteria and reductions in intestinal pH (Barragan-Fonseca et al., 2017; Spranghers et al., 2018), while chitin works as a prebiotic to support a diverse microbiome with lower abundance of pathogenic bacteria such as *E. coli* and *Salmonella* (Borrelli et al., 2017).

It was hypothesized that partially replacing SBM with BSFLM in plant-based nursery diets for pigs housed in non-disinfected pens would improve growth performance and fecal scores by modulating the diversity and distribution of intestinal microbiota. The objective was to determine the effect of partially replacing SBM with BSFLM in nursery pig diets on growth performance, fecal scores, and cecal mucosal microbial profile when weaned into non-disinfected pens.

## Materials and Methods

The experimental protocol was approved by the University of Guelph Animal Care Committee and followed Canadian Council on Animal Care guidelines (Canadian Council on Animal Care. 2009).

### Animals and Housing

One hundred, 21-d-old pigs obtained from the Arkell Swine Research station (Guelph, ON, Canada) were recruited for the study. Pigs were weaned (6.96 ± 0.23 kg BW) and placed into nursery pens assigning littermates to different pens (five pigs per pen), with sex distributed evenly among treatments. Each pen was equipped with a four-space stainless steel feeder and nipple drinker for ad libitum access to feed and water, respectively. For the first 48 h, the nursery room temperature was set between 20 and 21 °C. Thereafter, rooms were returned to normal conditions (29.5 °C initial temperature and reduced by 1.5 °C per week until reaching a constant temperature of 25 °C). Additionally, prior to weaning, the pens were cleaned with high-pressure, hot water without the use of detergents or disinfectants. This modification in temperature and sanitation procedure was employed to induce diarrhea in pigs, since this study was conducted in a high-health research facility where the incidence of post-weaning diarrhea is low.

### Dietary Treatments and Experimental Procedures

Each pen was randomly assigned to one of the four dietary treatments (*n* = 5): high-complexity (contained highly digestible animal proteins) with and without 3,000 ppm ZnO (HC** + **and HC−, respectively; representative of commercial diets), low-complexity, plant-based diet (corn- and SBM-based; LC), or LC with 30% inclusion of BSFLM to partially replace SBM (LCFL; Oreka Solutions, Markham, ON, Canada; Table 1). The BSFLM contained 88.4% dry matter, 5,035kcal/kg of gross energy, 42.5% crude protein, 1.87% Lys and 1.71% Arg, and 3,074 kcal/kg of NE (Crosbie et al., 2020). The HC + and HC− diets contained barley, dried whey, blood plasma, and oat groats, whereas the LC and LCFL diets contained greater amounts of corn and wheat, with the removal of dried whey, blood plasma, and oat groats. Additionally, LC diets had greater SBM inclusion than HC + and HC− diets, whereas LCFL included BSFLM to partially replace SBM and wheat. Each diet also contained 0.2% TiO_2_ as an indigestible marker for the determination of apparent total tract digestibility (ATTD) of energy and nutrients (Adeola, 2001). Experimental diets were fed for 14 d (phase I), followed by 4 wk of a common corn-SBM diet (phase II).

**Table 1.** Diet composition and calculated and analyzed nutrient contents of nursery diets (as-fed basis)

	Phase I^1^				Phase II
Item	HC+	HC−	LC	LCFL	Common diet
Ingredient, % (as-fed)					
Corn	15.05	15.35	47.10	54.93	46.32
Soybean meal, dehulled	10.80	10.80	31.80	8.00	30.00
Barley	25.00	25.00	—	—	—
Wheat	—	—	10.00	2.50	15.00
Whey, dried	20.00	20.00	**—**	—	—
Corn oil	5.00	5.00	5.40	—	—
Blood plasma^2^	4.50	4.50	—	—	—
Oat groats	10.00	10.00	—	—	—
Soybean isolate^3^	5.00	5.00	—	—	—
Black soldier fly larvae meal, full fat^4^	—	—	—	30.00	—
Zinc oxide^5^	0.30	—	—	—	—
Animal–vegetable fat blend	—	—	—	—	5.00
Monocalcium phosphate	1.94	1.94	1.73	1.65	0.97
Limestone	0.89	0.89	1.22	0.08	1.17
Salt	—	—	0.90	0.95	0.40
L-Lysine ∙ HCl	0.40	0.4	0.61	0.85	0.35
DL-Methionine	0.20	0.20	0.20	0.05	0.09
L-Threonine	0.12	0.12	0.22	0.17	0.10
L-Tryptophan	—	—	0.02	0.02	—
Mineral and vitamin premix^6^	0.60	0.60	0.60	0.60	0.60
Titanium dioxide	0.20	0.20	0.20	0.20	—
Total	100.00	100.00	100.00	100.00	100.00
Calculated nutrient composition, as-fed^7^					
Net energy, kcal/kg	2,691	2,699	2,613	2,717	2,633
Crude protein, %	21.03	21.28	21.02	22.34	20.64
Calcium, %	0.93	0.93	0.93	1.06	0.78
Phosphorus, %	0.94	0.95	0.80	0.75	0.65
Analyzed nutrient content, %, as-fed					
Crude protein	21.64	19.88	20.19	21.51	18.62
Calcium	0.81	0.77	0.81	0.93	0.77
Phosphorus	0.85	0.78	0.73	0.72	0.59
Lysine	1.73(1.57)^8^	1.62(1.57)	1.51(1.57)	1.76(1.61)	(1.33)
Threonine	0.97(0.99)	0.93(0.99)	0.80(0.98)	0.90(0.85)	(0.84)
Methionine	0.50(0.49)	0.48(0.49)	0.42(0.51)	0.43(0.43)	(0.40)
Arginine	1.02(1.20)	1.00(1.20)	1.10(1.31)	1.07(1.01)	(1.28)

^1^Dietary treatments: HC + and HC− = high-complexity diet that contained animal-derived protein sources with and without, respectively, 3,000 ppm added ZnO; LC = low-complexity diet containing only plant-based protein sources; LCFL = low-complexity diet with soybean meal partially replaced by full-fat black soldier fly larvae meal (BSFLM). Experimental diets were fed for 14 d after weaning; phase I. A common diet was fed to all pens for 4 weeks thereafter; phase II.

^2^AP920; manufactured by APC Nutrition Inc. (Ames, IA).

^3^Soybean isolate obtained from Old Mill Feeds (Elie, MB, Canada).

^4^Full-fat BSFLM obtained from Oreka Solutions (Markham, ON, Canada).

^5^Zinc Oxide obtained from Pestell Nutrition (New Hamburg, ON, Canada).

^6^Provided per kilogram of diet: 12,000 IU vitamin A as retinyl acetate, 1,299 IU vitamin D3 as cholecalciferol, 48 IU vitamin E as dl-α-tocopherol acetate, 3 mg vitamin K as menadione, 19 mg pantothenic acid, 6 mg riboflavin, 600 mg choline, 2.4 mg biotin, 18 mg Cu from CuSO4∙5H2O, 120 mg Fe from FeSO4, 24 mg Mn from MnSO4, 126 mg Zn from ZnO, 0.36 mg Se from Na2SeO3, and 0.6 mg I from KI (DSM Nutritional Products Canada Inc., Ayr, ON, Canada).

^7^Calculated based on the NRC (2012) ingredient values and Crosbie et al. (2021) for BSFLM ingredient values.

^8^Calculated amino acid contents are shown in parentheses.

Individual pig BW and per-pen feed disappearance were determined weekly for calculation of average daily gain (ADG), average daily feed intake (ADFI), and gain-to-feed ratio (G:F). For the first 7 d after weaning, on every second day, all pigs were scored on fecal consistency (0 = firm and dry to 3 = clear and water-like; Amezcua et al., 2007). Seven days after weaning, one pig per pen, that was identified as having diarrhea (fecal score of ≥ 1) was selected for necropsy. There were three pens (one pen from each HC+, LC, and LCFL) during the study that did not have any pigs with a fecal score ≥ 1, in which case a median BW pig was selected, that had at least one fecal score ≥ 1 on either day 1, 3, or 5 after weaning. Fecal swabs were collected from the selected pigs with a sterile cotton swab inserted 20 to 30 mm into the rectum and using small, gentle movements (circular and back- and forward) for a maximum of 2 min. Swabs were then placed in a sterile 15 mL falcon tube on ice for transport to the lab for enumeration of *E. coli* colony forming units (CFU). Pigs were euthanized with an intra-cardiac injection of Euthasol (3 mL; Virbac, TX), the entire gastrointestinal tract was excised, and full gut, liver, spleen, empty stomach, and empty small and large intestines were weighed. A 5-cm segment from the jejunum (~1.5 m distal to the ligament of Trietz) and ileum (~0.5 m proximal to the ileo-cecal junction) were collected, rinsed with saline (0.9% NaCl), and stored in 10% formalin until further evaluation. Jejunal and ileal samples were prepared for histomorphology measurements according to the procedures of Carleton et al. (1980). Measurements of the villus height (VH), villus width (VW), crypt depth, and crypt width (CW) were recorded for 10 replicates per parameter, per segment (i.e., jejunum and ileum), and per pig. Average values for each parameter were used to calculate villus height:crypt depth and absorptive capacity (AC). AC was calculated according to Kisielinski et al. (2002): equation 1


$$AC=\frac{(VW\times VH)+{\left(\frac{VW}{2}+\frac{CW}{2}\right)}^{2}-{\left(\frac{VW}{2}\right)}^{2}}{{\left(\frac{VW}{2}+\frac{CW}{2}\right)}^{2}}$$

Cecal mucosal samples were collected by isolating and emptying the cecum, rinsing with sterile phosphate-buffered saline, and then using a sterile swab gently brushing the mucosal surface to collect material. The swab was placed in a 1.5 mL Eppendorf tube on ice and then frozen at −80 °C until DNA extraction for cecal mucosal microbial community analysis. The ATTD of energy and nutrients were determined using uncontaminated fecal samples collected from the pen between days 5 and 7 and from individual pigs at the time of BW collection on day 7. Fecal samples were pooled per pen, stored at −20 °C, then freeze-dried and ground. Diet and fecal samples were analyzed for dry matter (AOAC, 2005; method 930.15), crude protein (AOAC, 2005; method 968.06), ash (AOAC, 2005; method 942.05), gross energy using a bomb calorimeter (IKA Calorimeter System C 5000; IKA Works Inc., Wilmington, NC) with benzoic acid as the calibration standard, and titanium content according to Myers et al. (2004) with modification as outlined in Christensen and Huber (2021). Diet samples were also analyzed for calcium and phosphorus using inductively coupled plasma mass spectrophotometry (AOAC, 2005; method 985.01; Agrifood Laboratories, Guelph, ON, Canada). Amino acid contents in the diets were analyzed using an ultra-performance liquid chromatography (Waters Corporation, Milford, MA) according to Cargo-Froom et al. (2019).

### 16S methods

Bacterial DNA was extracted using the QIAGEN DNeasy blood & tissue Kit following the manufacturer’s instructions. DNA concentrations were determined using Quant-iT Picogreen dsDNA Assay kits and dsDNA reagents through a fluorometer before being sent to the sequencing facility (MetagenomBio, Waterloo, Ontario) for 16S rRNA amplicon microbial community analysis. Using a set of universal primer (515FB: 5’- GTGYCAGCMGCCGCGGTAA, 806RB: 5’- GGACTACNVGGGTWTCTAAT), library DNA was sequenced targeting the V4 region of the 16S rRNA gene with MiSeq Reagent Kit v2 (2 × 250 cycles). The paired-end sequencing reads were preprocessed and assigned to amplicon sequence variants (ASVs) using the Divisive Amplicon Denoising Algorithm 2 (Callahan et al., 2016) implemented through the Quantitative Insights Into Microbial Ecology (QIIME2 version 2020.8) pipeline (Bolyen et al., 2019). Multiple sequence alignment was performed with MAFFT and FastTree 2 was conducted to generate and root a phylogenetic tree (Price et al., 2010). A pretrained Naive Bayes classifier trained on the Silva rRNA Database Project (version 138.1) was used to perform taxonomic classification analysis followed by removing reads that were assigned to non-bacterial domain, occurred only once (singleton), twice (doubleton), and ASVs with less than 10 % frequencies from the downstream analysis.

### Statistical Analyses

Statistical analyses for all parameters were conducted using the GLIMMIX procedure in SAS (University Edition; SAS Ins. Inc., Cary, NC), with treatment as the fixed effect. Means comparisons were conducted using the Tukey–Kramer test. A probability value (*P*) ≤ 0.05 was considered significant.

For the 16S rRNA microbial community analysis, the distribution of the taxa across the four dietary treatments (HC−, HC+, LC, and LCFL) was investigated using the “microbiome” package in R version 4.0.0. For the microbiota community composition analysis, the sequence reads were rarefied at a depth of 3,000. Alpha diversity was measured using the species observed and Shannon indices and the comparison between the dietary treatments was conducted using the Kruskal–Wallis test with Tukey Honest Significant Difference to control for multiple comparisons (Midway et al., 2020). Beta diversity was evaluated using Bray-Curtis dissimilarity and PERMANOVA analysis was performed to assess significance using the “adonis” function in the R “vegan” package (v.2.6-4). Differential abundance analysis was implemented at the aggregated genus level using the analysis of composition of Microbiomes with bias correction method (ANCOM-BC) (Lin and Peddada, 2020).

## Results

The analyzed composition of dietary crude protein was generally comparable to the calculated contents (Table 1). Analyzed dietary calcium and phosphorus contents were on average 16% and 12% less than calculated values for the experimental diets but were comparable to calculated values for the common, phase II diet. Analyzed total amino acid contents were also comparable to calculated values.

At weaning, pigs were assigned to treatments with BW evenly distributed (Table 2). Pig BW on days 7 and 14 and phase I ADG were lower for pigs fed LC and LCFL vs. pigs fed HC− and HC + (*P* < 0.001), which were not different. On day 42, pigs fed LCFL had lower BW than pigs fed HC− (*P *< 0.05), while intermediate values were observed for pigs fed HC + and LC. The ADG was not different between dietary treatments in phase II but over the entire nursery period, ADG was lower for pigs fed LCFL vs. HC− (*P *< 0.05), while intermediate values were observed for pigs fed HC + and LC. During phase I, ADFI was not different for pigs fed LC and LCFL, but lower than for pigs fed HC− (*P *< 0.001); pigs fed HC + had greater feed intake in phase I vs. all other treatment groups (*P *< 0.001). During phase II and over the entire nursery period, pigs fed LCFL had lower ADFI than pigs fed HC− and HC + (*P *< 0.001) and pigs fed HC− had greater ADFI than pigs fed HC + (*P *< 0.001); pigs fed LC had intermediate ADFI compared to pigs fed LCFL and HC+. During phase I, G:F was less for pigs fed LCFL vs. pigs fed HC− (*P *< 0.05), while intermediate values were observed for pigs fed LC and HC+. During phase II, G:F was greater for pigs fed LCFL and LC vs. pigs fed HC− (*P *< 0.01), while intermediate values were observed for HC+. Over the entire nursery period, G:F was not influenced by dietary treatment.

**Table 2.** Effect of full-fat black soldier fly larvae meal in low-complexity nursery diets on growth performance of pigs weaned into non-disinfected nursery pens

	Dietary treatment^1^					*P-*Value
	HC+	HC−	LC	LCFL	SEM^2^	Treatment
No.^3^	5	5	5	5		
Body weight, kg						
Day 0	7.25	7.44	7.19	7.10	0.17	0.146
Day 7	8.19^a^	8.20^a^	7.50^b^	7.32^b^	0.21	<0.001
Day 14	11.10^a^	11.04^a^	9.56^b^	9.10^b^	0.29	<0.001
Day 42	30.56^a,b^	30.80^a^	29.27^a,b^	28.22^b^	0.67	0.010
ADG, g						
Phase I	278^a^	259^a^	167^b^	137^b^	23	<0.001
Phase II	695	706	704	683	18	0.734
Overall	556^a,b^	557^a^	525^a,b^	503^b^	15	0.014
ADFI, g						
Phase I	336^a^	307^b^	241^c^	232^c^	8	<0.001
Phase II	1079^b^	1185^a^	1057^b,c^	1000^c^	20	<0.001
Overall	832^b^	892^a^	785^b,c^	744^c^	15	<0.001
G:F						
Phase I	0.83^a,b^	0.84^a^	0.70^a,b^	0.62^b^	0.06	0.010
Phase II	0.64^a,b^	0.60^b^	0.67^a^	0.68^a^	0.02	0.003
Overall	0.67	0.63	0.67	0.67	0.02	0.162

^1^Dietary treatments: HC + and HC− = high-complexity diet that contained animal-derived protein sources with and without, respectively, 3,000 ppm added ZnO; LC = low-complexity diet containing only plant-based protein sources; LCFL = low-complexity diet with soybean meal partially replaced by full-fat black soldier fly larvae meal. Experimental diets were fed for 14 d after weaning (phase I). A common diet was fed to all pens for 4 wk thereafter (phase II).

^2^Maximum value of standard error of the means.

^3^Number of pens evaluated per dietary treatment.

^a-c^Within a row, means without a common superscript differ, *P* < 0.05.

On day 3, fecal consistency score was greater (i.e., softer feces) for pigs fed LCFL vs. HC + (*P *< 0.01), while intermediate scores were observed for pigs fed HC− and LC (Table 3). Fecal consistency scores were not influenced by dietary treatment on days 1, 5, or 7. Fecal *E. coli* CFU were not different among pigs fed LCFL, LC, or HC−, but all were greater than pigs fed HC + (*P *< 0.001).

**Table 3.** Effect of full-fat black soldier fly larvae meal in low-complexity nursery diets on fecal consistency scores and fecal *E. coli* colony forming units (CFU/g) after weaning pigs into non-disinfected nursery pens

	Dietary treatment^1^					*P-*Value
	HC+	HC−	LC	LCFL	SEM^2^	Treatment
No.^3^	5	5	5	5		
Fecal consistency score^4^						
Day 1	0.05	0.07	0.04	0.04	0.05	0.965
Day 3	0.65^b^	1.67^a^^,^^b^	1.08^a^^,^^b^	1.46^a^	0.17	0.006
Day 5	1.20	1.23	1.20	1.24	0.18	0.997
Day 7	0.70	0.80	0.54	0.92	0.16	0.278
*E. coli*, CFU/g log10 ^5^	6.66^b^	8.61^a^	8.70^a^	8.58^a^	0.26	<0.001

^1^Dietary treatments: HC + and HC− = high-complexity diet that contained animal-derived protein sources with and without, respectively, 3,000 ppm added ZnO; LC = low-complexity diet containing only plant-based protein sources; LCFL = low-complexity diet with soybean meal partially replaced by full-fat black soldier fly larvae meal. Experimental diets were fed for 14 d after weaning (phase I).

^2^Maximum value of standard error of the means.

^3^Number of pens evaluated per dietary treatment.

^4^Fecal consistency score: 0-firm and dry feces, 1-soft and pasty feces, 2-yellowish fluid feces, 3-clear, water-like feces.

^5^ *E. coli* colony forming units were conducted on day 7 fecal samples.

^a,b^Within a row, means without a common superscript differ, *P* < 0.05.

### Organ Weights, Nutrient Digestibility, and Intestinal Histomorphology

Full gut, spleen, empty stomach, and empty small and large intestine weights were not affected by dietary treatment on day 7 (Table 4). Live BW was influenced by dietary treatment (*P *< 0.05), but no statistical differences were detected among means using the Tukey test. Pigs fed LCFL and LC had lower liver weights vs. pigs fed HC + (*P *< 0.001), while intermediate values were observed for pigs fed HC−. Organic matter and gross energy ATTD and digestible energy were less for LCFL vs. HC− (*P *< 0.01), while intermediate values were observed for HC + and LC (Table 4). Of the histomorphology measurements collected for jejunum and ileum, only jejunal AC was affected by dietary treatment, with LCFL and LC having less AC than HC− and HC + (*P *< 0.05; Table 5), which were not different.

**Table 4.** Effect of full-fat black soldier fly larvae meal in low-complexity nursery diets on organ weights and apparent total tract component digestibility (ATTD) 7 d after weaning pigs into non-disinfected nursery pens

	Dietary treatment^1^					*P-*Value
	HC+	HC−	LC	LCFL	SEM^2^	Treatment
No.^3^	5	5	5	5		
Live body weight, kg	7.93	7.74	6.96	7.16	0.26	0.048
Organ weight, g						
Full gut	952	932	797	838	86	0.533
Liver	195^a^	177^a^^,^^b^	152^b^	156^b^	6	<0.001
Spleen	34	29	25	30	3	0.160
Empty stomach	48	50	46	44	4	0.729
Empty small intestine	313	333	278	280	23	0.299
Empty large intestine	95	103	98	83	7	0.306
ATTD, %						
Organic matter	82.7^a^^,^^b^	83.8^a^	79.0^a^^,^^b^	77.3^b^	2.0	0.006
Ash	61.8^a^	60.7^a^^,^^b^	48.7^b^	49.5^a^^,^^b^	3.1	0.013
Gross energy	79.5^a^^,^^b^	80.5^a^	74.7^a^^,^^b^	71.5^b^	1.5	0.002
Digestible energy						
Kcal/kg	3,661^a^^,^^b^	3,672^a^	3,434^a^^,^^b^	3,381^b^	71	0.018

^1^Dietary treatments: HC + and HC− = high-complexity diet that contained animal-derived protein sources with and without, respectively, 3,000 ppm added ZnO; LC = low-complexity diet containing only plant-based protein sources; LCFL = low-complexity diet with soybean meal partially replaced by full-fat black soldier fly larvae meal. Experimental diets were fed for 14 d after weaning (phase I).

^2^Maximum value of standard error of the means.

^3^Number of pens evaluated per dietary treatment.

^a,b^Within a row, means without a common superscript differ, *P* < 0.05.

**Table 5.** Effect of full-fat black soldier fly larvae meal in low-complexity nursery diets on jejunal and ileal morphology 7 d after weaning pigs into non-disinfected nursery pens

	Dietary treatment^1^					*P-*Value
	HC+	HC−	LC	LCFL	SEM^2^	Treatment
No.^3^	5	5	5	5		
Jejunum						
Villus height (VH), μm	472	466	345	392	59	0.315
Villus width, μm	117	104	141	133	16	0.354
Crypt depth (CD), μm	246	315	255	249	44	0.647
Crypt width, μm	44	44	104	81	23	0.173
VH:CD	2.13	1.58	1.43	1.60	0.31	0.332
Absorptive capacity, μm^2^	9.03^a^	9.31^a^	4.72^b^	5.87^b^	1.14	0.016
Ileum						
Villus height, μm	410	337	352	367	42	0.643
Villus width, μm	105	115	113	129	15	0.504
Crypt depth, μm	211	227	233	212	31	0.948
Crypt width, μm	42	53	76	132	32	0.236
VH:CD	1.99	1.57	1.65	1.97	0.28	0.641
Absorptive capacity, μm^2^	8.46	6.31	6.67	4.90	1.18	0.243

^1^Dietary treatments: HC + and HC− = high-complexity diet that contained animal-derived protein sources with and without, respectively, 3,000 ppm added ZnO; LC = low-complexity diet containing only plant-based protein sources; LCFL = low-complexity diet with soybean meal partially replaced by full-fat black soldier fly larvae meal. Experimental diets were fed for 14 d after weaning (phase I).

^2^Maximum value of standard error of the means.

^3^Number of pigs evaluated per dietary treatment.

^a,b^Within a row, means without a common superscript differ, *P* < 0.05.

### Cecal Mucosal Microbiota Composition

**Figure 1.**
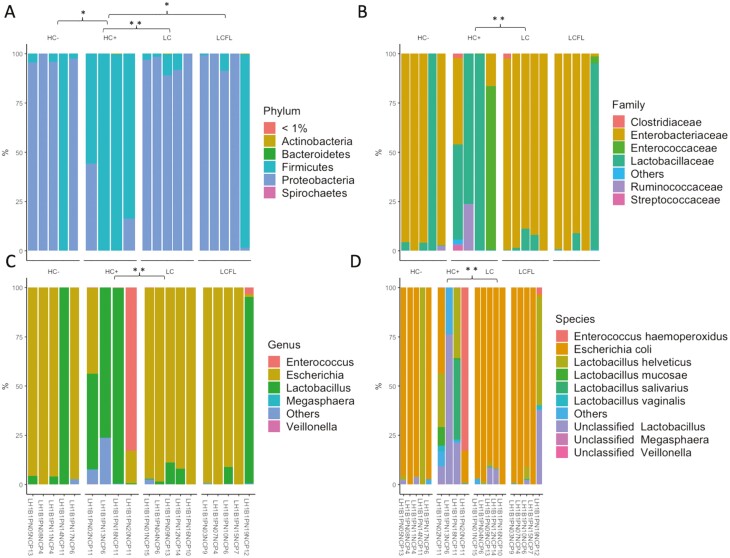
Relative abundance of top taxa among cecal mucosal samples on day 7 after weaning at the phyla (panel A), family (panel B), genus (panel C), and species (panel D) levels. Asterisks show significance values (* *P* < 0.05 and ** *P* < 0.01). Dietary treatments: HC + and HC− = high-complexity diet that contained animal-derived protein sources with and without, respectively, 3,000 ppm added ZnO; LC = low-complexity diet containing only plant-based protein sources; LCFL = low-complexity diet with soybean meal partially replaced by full-fat black soldier fly larvae meal.

The cecal mucosal microbial community comprised primarily five phyla, *Actinobacteria, Bacteroidetes, Firmicutes, Proteobacteria*, and *Spirochaetes*, while *Cyanobacteria, Deferribacteres,* and *Tenericutes* accounted for less than 1% relative abundance in all samples (Figure 1A). At the phylum level, there were lower relative proportions of *Firmicutes* in HC− (*P* < 0.05), LC (*P* < 0.01), and LCFL (*P* < 0.05) compared to pigs fed HC+. *Clostridiaceae, Enterobacteriaceae, Enterococcaceae, Lactobacillaceae, Ruminococcaceae,* and *Streptococcaceae* were found to be the top six families, which accounted for ~78% of the total sequences (Figure 1B). At the family level, the relative proportion of *Lactobacillaceae* was lower for pigs fed LC vs. HC + (*P* < 0.01). The most abundant genera were *Escherichia*, *Lactobacillus, and Enterococcus* (Figure 1C). The most abundant species were *Escherichia coli, Lactobacillus helveticus, Lactobacillus mucosae, Enterococcus haemoperoxidus,* and *unclassified Lactobacillus* (Figure 1D). At the genus and species levels, the relative proportion of *Lactobacillus* was lower for pigs fed LC vs. HC + (*P* < 0.01).

**Figure 2.**
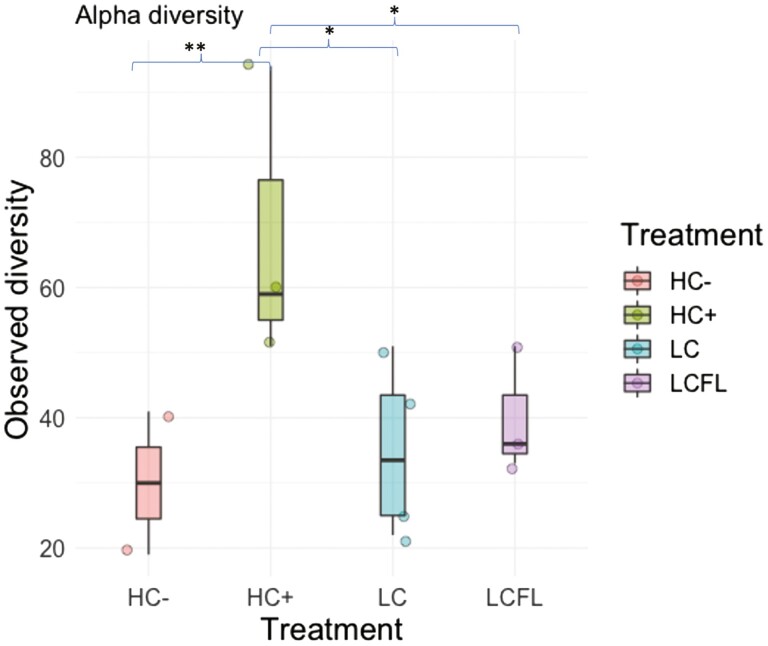
Alpha diversity analysis among the rarefied samples from the cecal mucosa on day 7 after weaning. Asterisks show significance values (* *P* < 0.05 and ** *P* < 0.01). Dietary treatments: HC + and HC− = high-complexity diet that contained animal-derived protein sources with and without, respectively, 3,000 ppm added ZnO; LC = low-complexity diet containing only plant-based protein sources; LCFL = low-complexity diet with soybean meal partially replaced by full-fat black soldier fly larvae meal.

**Figure 3.**
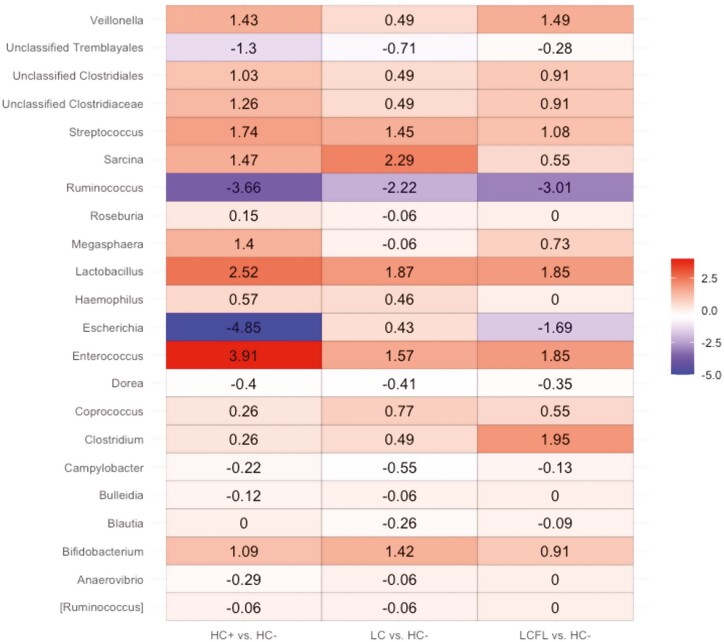
Differentially abundant taxa displayed by color intensity. Data are represented by absolute log2 fold-change (effect size) and 95% confidence interval (Bonferroni adjusted) derived from analysis of compositions of microbiomes with bias correction (ANCOM-BC). Y-axis represents the significantly different abundant ASVs at the genus-level along with the log2 fold change (X-axis). Positive values indicate that the taxon is highly abundant while negative values represent less abundant taxa across the four dietary treatments. Dietary treatments: HC + and HC− = high-complexity diet that contained animal-derived protein sources with and without, respectively, 3,000 ppm added ZnO; LC = low-complexity diet containing only plant-based protein sources; LCFL = low-complexity diet with soybean meal partially replaced by full-fat black soldier fly larvae meal.

Alpha diversity analysis using the Observed and Shannon diversity indices identified significantly different richness across the four dietary treatments. Pigs fed HC− (*P* < 0.01), LC (*P* < 0.05), and LCFL (*P* < 0.05) had lower richness compared to HC + (Figure 2). Bray-Curtis dissimilarity distance identified different community compositions of pigs fed LC compared to HC + (tendency; *P* = 0.053) and LCFL (tendency; *P* = 0.097). Overall, the analysis outcomes suggest that HC + and LCFL groups showed more similar microbial composition profiles vs. the other treatment groups. Furthermore, the analysis of the differences at the genus level revealed 22 differential ASVs (*P* < 0.05) between the four dietary treatments (Figure 3). Notably, HC + and LCFL had low abundance of *Escherichia* and *Ruminococcus* and high abundance of *Enterococcus* and *Lactobacillus* compared to pigs fed the HC− treatment.

## Discussion

The objective of the current study was to determine the effect of partially replacing SBM with BSFLM in plant-based nursery pig diets on growth performance, fecal scores, and cecal mucosal microbial profile when weaned into non-disinfected pens. Based on this experiment, pigs fed diets containing only plant-based proteins or diets that partially replaced SBM with BSFLM during the first 2 wk after weaning were not able to achieve growth performance (ADG or ADFI) comparable to pigs fed diets containing highly digestible animal protein sources, with or without pharmacological ZnO. Previous work has shown that pigs fed low-complexity diets after weaning had reduced growth compared to pigs fed high-complexity diets (Christensen and Huber, 2021); however, in the current study, no improvements in growth performance were observed when BSFLM was used to partially replace SBM. Additionally, improvements in growth performance were also expected with the addition of pharmacological ZnO to HC diets, as this has been reported in previous studies (e.g., Woodworth et al., 2015). Moreover, even after consuming a common diet for 4 wk, pigs fed diets with BSFLM were not able to achieve compensatory growth within the nursery period and still had nursery exit BW less than pigs fed HC−. Previous work has shown improvements in body weight 1 wk after weaning when BSFLM was included in the diet at 7.4% to partially replace animal protein sources (Crosbie et al., 2021). When the inclusion of BSFLM was increased to 14.8%; however, body weight was negatively affected after phase I (Crosbie et al., 2021), but others observed that inclusion of BSFLM up to 19.1% could be used in nursery pig diets, with minimal effects on growth performance (Håkenåsen et al., 2021). Based on the total analyzed dietary amino acid contents, estimated standardized ileal digestibility of amino acids (NRC, 2012), and reduced feed intake for pigs fed the plant-based diets in the current study, Lys intake may have limited protein deposition (lean growth) during phase I as Lys intake during this phase was lower than estimated requirements (standardized ileal digestible Lys intake g/day: LC = 3.6; LCFL = 4.0; estimated requirements = 4.8; NRC, 2012). It is possible that BSFLM is better suited for lower inclusion levels in the diet than what was used in this current study (30% inclusion). Therefore, BSFLM may be more effective when included below 7% in nursery diets.

The inclusion of BSFLM to partially replace SBM in a plant-based nursery diet in the current experiment was also not able to influence the incidence of soft feces or fecal *E. coli* CFU after weaning. The BSFLM is a source of highly digestible amino acids for pigs (Crosbie et al., 2020), but it also contains chitin and MCFA, which have prebiotic and antimicrobial properties (Spranghers et al., 2018). The MCFA have also been shown to have anti-inflammatory characteristics that can mitigate villus atrophy in nursery pigs (Gebhardt et al., 2020). Thus, it was expected that pigs provided the diet with BSFLM would have less fecal *E. coli* CFU and improved fecal scores and intestinal histomorphology characteristics, but the opposite was observed on day 3 for fecal scores and day 7 for *E. coli* CFU and jejunal AC (vs. pigs fed HC+). Therefore, it appears that the functional components in full-fat BSFLM did not protect pigs from weaning- and/or SBM-induced losses of jejunal AC and did not reduce fecal shedding of *E. coli*, even when provided at high inclusion levels.

The current experiment also demonstrated that pharmacological ZnO is effective at improving feed intake and fecal scores and reducing fecal *E. coli* shedding after weaning, as have many others (Yu et al., 2017; da Silva et al., 2021; Christensen et al., 2022). It is well known that pharmacological ZnO increases blood ghrelin concentration, which promotes feed intake thereby reducing villus atrophy and intestinal dysfunction after weaning (Yin et al., 2009). The current study also demonstrated that pigs fed the diet containing pharmacological ZnO had greater alpha diversity and less relative abundance of *E. coli* in favor of bacteria from the genus *Lactobacillus* in the cecal mucosal microbiome. da Silva et al. (2021) reported ZnO increased bacteria abundance in the genus *Roseburia,* which is generally considered beneficial as it produces butyrate, a beneficial short-chain fatty acid (Anwar et al., 2017). Conversely, in the current study, the inclusion of BSFLM did not modulate the cecal mucosal microbiota to the same extent. Others demonstrated that feeding BSFLM to replace SBM in growing pig diets increased the Shannon diversity index for the microbiota in the jejunum whilst no difference was observed for the microbiota in the ileum (Kar et al., 2021) indicating that BSFLM may have less influence on distal intestinal microbiota. Indeed, Håkenåsen et al. (2021) also observed no differences in alpha diversity indices in the colon of nursery pigs feed increasing levels of BSFLM up to 19.06% of the diet. However, in the aforementioned study, small intestinal morphology and incidence of diarrhea were not assessed. Additionally, our current experiment found that pigs fed LCFL had greater relative abundance of Lactobacillus than pigs fed HC−. This effect on Lactobacillus abundance was also observed in previous studies when pigs were fed diets containing BSFLM (Håkenåsen et al., 2021). Therefore, the findings from the current study are in line with previous research, such that the inclusion of ZnO resulted in a greater diversity in the cecal mucosal microbial profile; however, the inclusion of BSFLM only marginally influenced alpha diversity at the cecal mucosa. Differences in fiber source can also influence microbiota and fecal consistency scores (Chen et al., 2014), however, this effect was not observed in the current study (i.e., there was no difference in fecal consistency scores or fecal E. coil CFU between HC−, LC, and LCFL). It should be noted that sample sizes for the 16S rRNA microbial community analysis may have been a limiting factor for this evaluation; however, as fecal score improvements were not observed at this inclusion level, a more comprehensive analysis was not justified.

Although BSFLM can be supplied as an alternative protein source for nursery pigs, this study did not find any benefits of providing BSFLM over SBM for 14 d after weaning. In general, pigs fed diets containing animal-based protein sources and pharmacological ZnO had superior growth performance, driven by increased feed intake and improved cecal microbial diversity which corresponded to reduced incidence of diarrhea. Conversely, pigs fed plant-based diets with BSFLM had reduced growth during phase I and were unable to exhibit compensatory growth during phase II. Although LCFL reduced the abundance of Escherichia vs. pigs fed HC−, the cecal mucosal samples were still more heavily dominated by *E. coli* than for pigs fed HC+. Therefore, partially replacing SBM with BSFLM in plant-based nursery diets for pigs housed in non-disinfected pens did not modulate the cecal mucosal microbial communities to a great enough extent to elicit growth performance and fecal consistency improvements, which is contrary to the effects of highly digestible diets containing animal proteins and ZnO.
